# "Organ transplantation was a second chance given to me": a qualitative study of the experiences of solid organ transplantation patients

**DOI:** 10.1590/1806-9282.20241640

**Published:** 2025-05-02

**Authors:** Yeliz Sürme, Eda Albayrak Günday, Handan Topan

**Affiliations:** 1 Erciyes University, Faculty of Health Sciences, Department of Surgery Nursing – Kayseri, Turkey.; 2 Erciyes University, Faculty of Health Sciences, Department of Mental Health and Diseases Nursing – Kayseri, Turkey.

**Keywords:** Organ grafting, Requirements, Transplantation, Qualitative research

## Abstract

**OBJECTIVE:**

The aim of this study was to qualitatively examine the needs of patients undergoing organ transplantation.

**METHODS:**

The sample of the study consisted of 17 patients. Data were evaluated using Colaizzi's seven-stage content analysis method.

**RESULTS:**

Two themes such as returning to daily life after organ transplantation and difficulties experienced after organ transplantation were determined.

**CONCLUSION:**

Individuals experienced physiological problems and fear of rejection after organ transplantation. Moreover, patients who experienced rejection fell into despair. However, their quality of life increases in the long term. Besides, individuals could not receive social support during transplantation.

## INTRODUCTION

Currently, there is no treatment option available other than organ transplantation for resuscitation of patients^1,2^. Organ transplants vary from country to country around the world but are obtained from cadaver donors. However, there are not enough organs to meet the need, and the number of patients waiting for transplantation is increasing day by day^3-5^. Turkey ranks first in the world among the countries that receive organ transplants from living donors^6^.

In the transplantation process, providing the best conditions for the donor and the recipient, improving the quality of life in the later period, and meeting the information needs are as important as the successful transplantation of the organ^2,4^. Although the health-related quality of life increases after transplantation, the level of the quality of life of healthy people cannot be reached^4,7^. After transplantation, individuals experience many problems, including pain because of surgery, nausea, vomiting, susceptibility to infections, loss of concentration, hair loss, loss of sexual desire, and depressive feelings because of immunosuppressive drugs. These symptoms can negatively affect the individual's quality of life and impair their adherence to treatment^7,8^. All these may cause feelings such as hopelessness, anxiety, and fear after transplantation, which may negatively affect the patients’ compliance with treatment.

Transplantation is quite challenging for patients. Hence, this qualitative study was conducted to examine the needs of patients who underwent solid organ transplantation.

## METHODS

### Type of research

This research is a phenomenological qualitative study carried out to examine the needs of patients who underwent solid organ transplantation.

Qualitative research is especially recommended when complex issues need to be explored in depth. Phenomenology is a qualitative research method used in the in-depth investigation of experiences, enabling individuals to express their perspectives and perceptions^9^.

### Participants

Social media increases communication and interactions about health. Today, people seek answers to issues related to their health by using social media tools, and health professionals answer the questions of individuals using these tools^10^. Facebook is one of the most popular social media platforms^11^.

The universe of the research consisted of patients in groups created about organ transplantation on Facebook. The purposive sampling method was used in the study. The sample of the study consisted of patients who had stated in these Facebook groups that they had undergone transplantation, had posts to search for post-transplant information, and met the inclusion criteria of the study.

The inclusion criteria for the study were as follows: (1) Patients who are included in groups about organ transplantation on Facebook; (2) solid organ transplant patients; and (3) those aged 18 years and older and agreed to participate in the study.

Qualitative studies do not have a fixed sample size and interviews should continue until data saturation is reached^12,13^. The present study was completed with 17 patients whose data started to repeat, and data saturation was reached.

### Data collection

The data were collected via mobile phone by the researcher (EAG), who is a psychiatric nurse specialist and experienced in qualitative research, between April 15 and June 15, 2022, using an introductory information form and a semi-structured interview form.

Introductory information form: In this form, there are 12 questions regarding introductory information.

Semi-structured interview form: This form consists of four open-ended questions developed by the authors after reviewing the relevant literature^8^ (Table 1).

**Table 1:** Semi-structured interview questions.

What has changed in your life after organ transplantation?
What are the difficulties/problems you experience after organ transplantation?
How did the pandemic period affect your disease process as a transplanted individual?
What are the problems you encountered as an organ transplant individual during the pandemic period?

First, a private message was sent to individuals who stated that they had undergone transplantation and had been seeking information in the aforementioned Facebook groups. The purpose of the study was explained to these individuals and they were invited to the study. The interviews were audio recorded with the consent of the participants. The interviews lasted an average of 30–35 min. Incomprehensible questions and answers were repeated. A single interview was conducted with each participant.

### Rigor and trustworthiness

The reliability of the data was obtained based on the strategies determined by Jiggins Colorafi and Evans^14^. These strategies are consistency, confirmability, reliability, and transferability. For consistency, all interviews were conducted by the same researcher. For confirmability, the data were transferred to a computer, stored, and analyzed independently by two researchers (YS and EAG). For the reliability of the study, the participants were encouraged to freely express their views at the beginning of the interview. An expert opinion was taken for the suitability of the interview forms. In addition, two experts experienced in qualitative research were consulted in order to confirm whether the sub-themes given under the conceptual theme reached in the research represent the mentioned conceptual category. Experts agreed that the sub-themes represent the themes. Participant confirmation was obtained to ensure reliability. The study is transferrable since data saturation is ensured. In this study, sample selection, participant characteristics, and how the study was conducted were explained in detail to ensure transferability.

### Research team

The research team consisted of three researchers who are academicians. The first researcher has a doctorate in surgical nursing and has performed studies on organ transplantation. The second researcher has a doctorate in psychiatric nursing, and the third researcher who conducted the interviews was a psychiatric nurse who ensured the sustainability of effective communication and interviews with the participants. All the researchers have scientific research experience in qualitative research.

### Data analysis

Data were evaluated using Colaizzi's seven-stage content analysis method^15^. The interviews were transcribed word for word. All data were read multiple times by two researchers, and important statements were underlined. Meanings were formed from important expressions. Important statements were decided based on their frequent repetition by the patients and their occurrence in the literature. Frequently repeated common statements were grouped and categorized, and themes and sub-themes were created by revealing the relationships between the categories. The created themes and sub-themes were integrated with the patients’ experiences. The basic framework of the experiences of the patients who had undergone transplant surgery was established. Finally, the data obtained from the interviews were summarized and the patients were asked whether they wanted to add any statements regarding the accuracy of the data, but they did not make any additions.

The themes were supported by direct quotations^12^. The Consolidated Criteria for Reporting Qualitative Research checklist was followed during the study^16^.

### Ethical consideration

Ethics committee approval (2022-122) was obtained from Erciyes University Social and Human Sciences, and verbal informed consent was obtained from the patients.

## RESULTS

In this study, 76.5% of the patients were male, 70.5% were married, and their mean age was 45.52±11.97 years. Notably, 70.5% of the patients had a liver transplant, all patients received transplants from a living donor, and 64.7% of them received organs from their relatives (Table 2).

**Table 2:** Demographic variables of the patients (n=17).

Demographic variables		n	%
Gender			
	Female	4	23.5
	Male	13	76.5
Age (mean±SD [min–max])		45.52±11.97 (30–67)	
Waiting time for transplantation		1.76±2.18 (0–6 years)	
Average time after transplantation		7.52±6.04 (2–19 years)	
Marital status			
	Married	12	70.5
	Single	5	29.5
Occupation			
	Worker	7	41.1
	Retired	6	35.2
	Officer	2	11.8
	Not working	2	11.8
Transplanted organ			
	Kidney	5	29.5
	Liver	12	70.5
Donor type*			
	Relatives	11	64.7
	Unknown person	6	36.3

* All donors were living donors.

SD: standard deviation.

Three main themes and five sub-themes were determined and are summarized in Table 3.

**Table 3:** Themes and sub-themes.

Themes	Sub-themes
Return to daily life after organ transplantation	Restoring health Change of environment
Difficulties experienced after organ transplantation	Physiological problems Rejection and fear of rejection Fear that something will happen to the donor

### Theme 1. Return to daily life after organ transplantation

Some of the patients stated that they regained their old health and life after transplantation. Some of the patients stated that they changed their environment after transplantation.

#### Sub-theme 1.1. Restoring health

Some of the patients stated that they regained their old health, their appetite had increased, and they paid more attention to their health than before.

"*I started living healthy. I cut out everything unhealthy from my life. The transplant was a second chance given to me*" *(P-8, F, 42 years)*.

#### Sub-theme 1.2. Change of environment

Some of the patients mentioned that they kept a distance from their relatives, who stayed away from them in order not to donate organs.

"*My environment has changed. When I was in such trouble, my relatives were not with me. When you are in trouble, you expect support from your relatives*" *(P-7, M, 58 years)*.

### Theme 2. Difficulties experienced after organ transplantation

Some of the patients stated that they had some physical problems after transplantation, most of them experienced fear of rejection, and some of them stated that they experienced rejection. Most of the patients stated that they were worried about the health of their donor.

#### Sub-theme 2.1. Physiological problems

Some of the patients stated that they experienced physiological problems, such as edema, stenosis, and long-term drains after the transplant surgery.

"*The complications of the surgery made me very exhausted. My drains were left too long. There have been strictures. It forced me for 9 months*" *(P-11, M, 59 years)*.

#### Sub-theme 2.2. Rejection and fear of rejection

Some of the patients stated that rejection developed after the organ transplant surgery and they needed to be transplanted again, while some of them stated that they experienced fear because of the risk of rejection after the transplant.

"*There was a protein leak in 2015. I had COVID-19 and a lung infection twice. This caused my kidney to fail and reject. I had the second COVID-19 very hard. I need to be transplanted again*" *(P-16, M, 41 years)*.

Most of the patients stated that they were afraid of experiencing rejection.

#### Sub-theme 2.3. Fear that something will happen to the donor

Some of the patients stated that they were worried about the health of their relatives who donated organs for organ transplantation, and they felt responsible.

"*I had a very stressful surgery. I was terrified. I was anxious that something would happen to the donor of the liver. I have established close relations with the donor*" *(P-7, M, 58 years)*.

## DISCUSSION

Although significant progress has been made in recent years with the development of technology, drugs, and surgical techniques, organ supply remains the primary problem^17^. In this study, it was found that 17 participants had similar experiences in the post-transplantation period, such as paying attention to a healthy life, fear of something happening to donors, and environmental changes.

The period with the highest incidence of infection in the post-transplantation period is between the first and sixth months^17,18^. In this study, individuals experienced physiological problems, such as edema and stenosis, in the early post-transplantation period, but their quality of life increased afterward, and individuals saw transplantation as a second chance and tried to lead a more regular life. Studies have shown that individuals experience problems, such as infection, especially in the first months after transplantation^2,19^. In the study conducted by Dinkçi et al., individuals experienced problems, especially in the first month, and their condition improved toward the sixth month^18^.

Organ transplantation is a process where hard experiences are experienced by the individuals before, during, and after transplantation. Individuals can be more sensitive during this period and seek more support from their relatives^6^. In this study, patients kept a distance from their relatives, who stayed away from them in order not to donate organs.

Fear of organ rejection, acceptance of the new organ, and psychosocial support systems are very important in coping with the disease^20^. Individuals may have concerns about loss of function, medical care costs, and organ rejection^2,19,20^. In this study, some of the patients stated that rejection developed after the organ transplant surgery and they needed to be transplanted again, while others stated that they had a fear of and hopelessness because of rejection after transplantation. The results are in line with the literature^2,19,20^.

In a qualitative study, it has been stated that individuals experience feelings of gratitude and guilt toward the organ donor^21^. Similarly, in this study, some of the patients stated that they were worried about the health of their relatives, who donated organs for organ transplantation, and that they felt responsible.

## CONCLUSION

After organ transplantation, individuals experienced physiological problems, such as edema, stenosis, and long drains, and some symptoms such as rejection and fear of rejection appeared, but their health was regained in the long term. Some individuals could not get support from their relatives during transplantation.
